# Supplementary material for: Multi-compartmental diversification of neutralizing antibody lineages dissected in SARS-CoV-2 spike-immunized macaques
Source: Nat Commun. 2024 Jul 27;15:6338. doi: 10.1038/s41467-024-50286-0 (PMC11283548; doi:10.1038/s41467-024-50286-0)
Supplement: Supplementary file 1 — Supplementary Information [file 41467_2024_50286_MOESM1_ESM.pdf]

Supplementary Information for

"Multi-compartmental diversification of neutralizing antibody lineages dissected in SARS-CoV-2 spike-immunized macaques"

Marco Mandolesi[1,*], Hrishikesh Das[2], Liset de Vries[1], Yiqiu Yang[1], Changil Kim[1], Manojj Dhinakaran[1], Xaquin Castro Dopico[1], Julian Fischbach[1], Sungyong Kim[1], Mariia V. Guryleva[1], Monika Àdori[1], Mark Chernyshev[1], Aron Stålmarck[1], Leo Hanke[1], Gerald M. McInerney[1], Daniel J. Sheward[1], Martin Corcoran[1], B. Martin Hällberg[2], Ben Murrell[1,#], Gunilla B. Karlsson Hedestam[1,#,*].

[1]Department of Microbiology, Tumor and Cell Biology, Karolinska Institutet, Stockholm, Sweden, [2]Department of Cell and Molecular Biology, Karolinska Institutet, Stockholm, Sweden

#Equal contribution

*Correspondence:

Marco Mandolesi, Department of Microbiology, Tumor and Cell Biology, Karolinska Institutet, S-171 77 Stockholm, Sweden. E-mail: marco.mandolesi@ki.se

Gunilla B. Karlsson Hedestam, Department of Microbiology, Tumor and Cell Biology, Karolinska Institutet, S-171 77 Stockholm, Sweden. E-mail: gunilla.karlsson.hedestam@ki.se

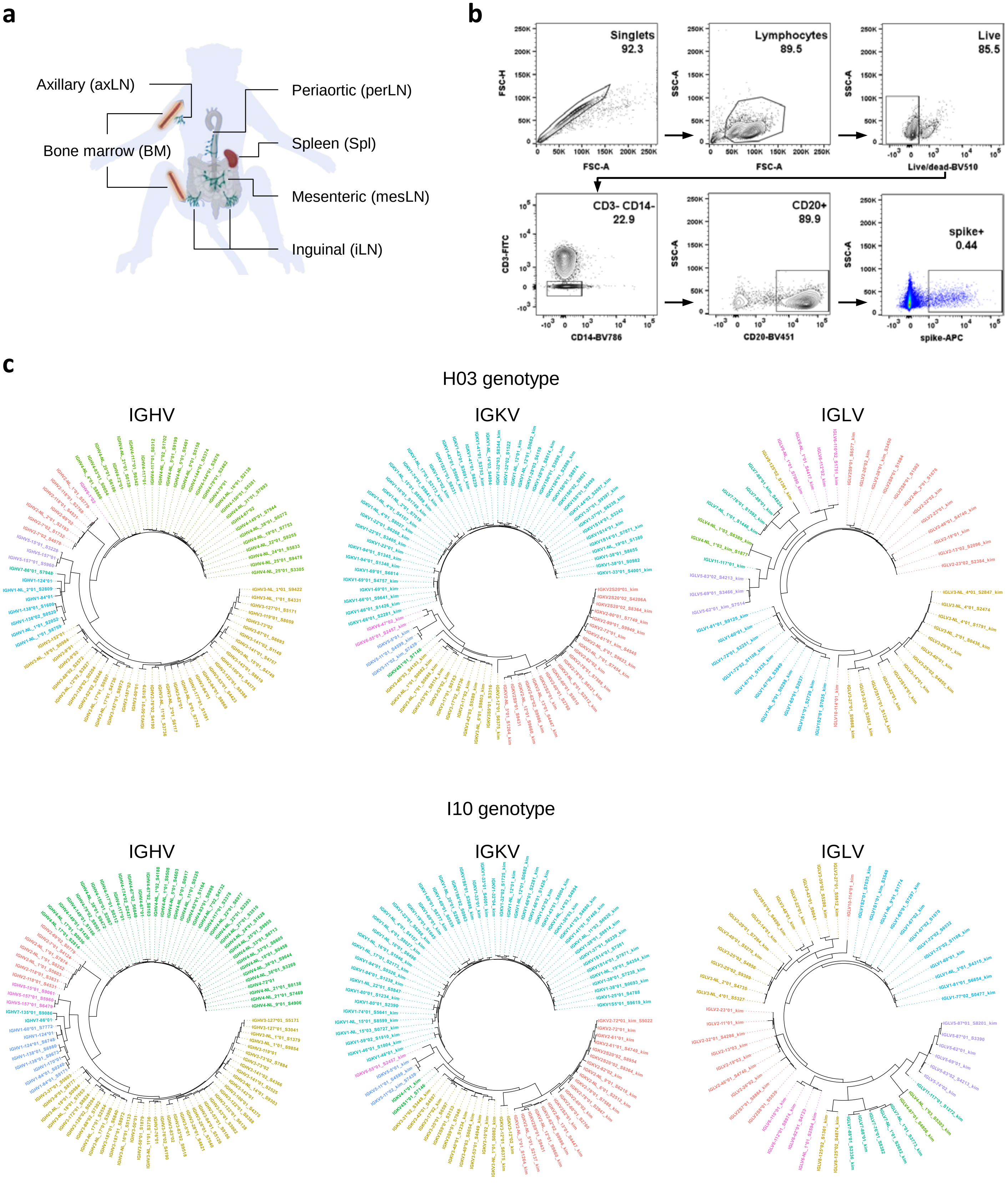

Supplementary Fig 1. Overview of sampled compartments, sorting strategy and germline heavy and light chain V alleles in H03 and I10. a Schematic representation of samples collected during the immunization regimen and at necropsy. Figure created with BioRender.com **b** Representative gating strategy (from iLN-R1) to sort spike-binding B cells. **c** *IGHV*, *IGKV* and *IGLV* germline alleles identified in H03 and I10 with alternating colors based on gene family.

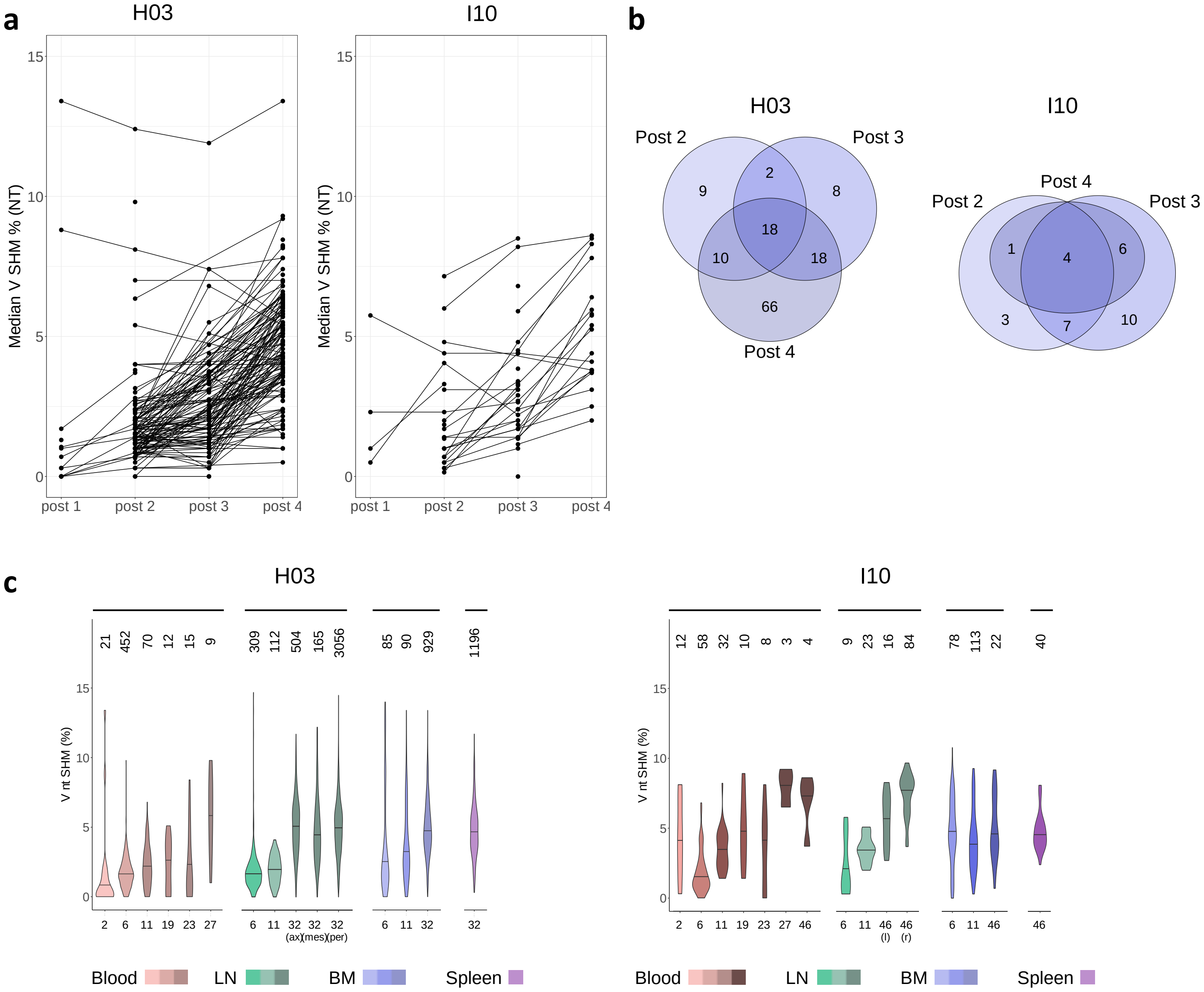

Supplementary Fig 2. Lineage tracing summary of H03 and I10. a Median *IGHV* gene SHM (% at the nucleotide level) per lineage and time point for H03 (left) and I10 (right). Source data are provided as a Source Data file. **b** Summary of repertoire overlap between BM samples collected at different immunization time points displayed as Venn diagram. The number of unique and shared lineages is displayed for H03 (left) and I10 (right). **c** SHM distribution per compartment for traced sequences. The samples are color coded with red, blue, green, and purple for blood, BM, LN, and spleen, respectively, with darker shades for increasing weeks. The number of sequences contributing to the analysis are shown over each violin plot for H03 (left) and I10 (right). Median values are represented in each plot. Source data are provided as a Source Data file.

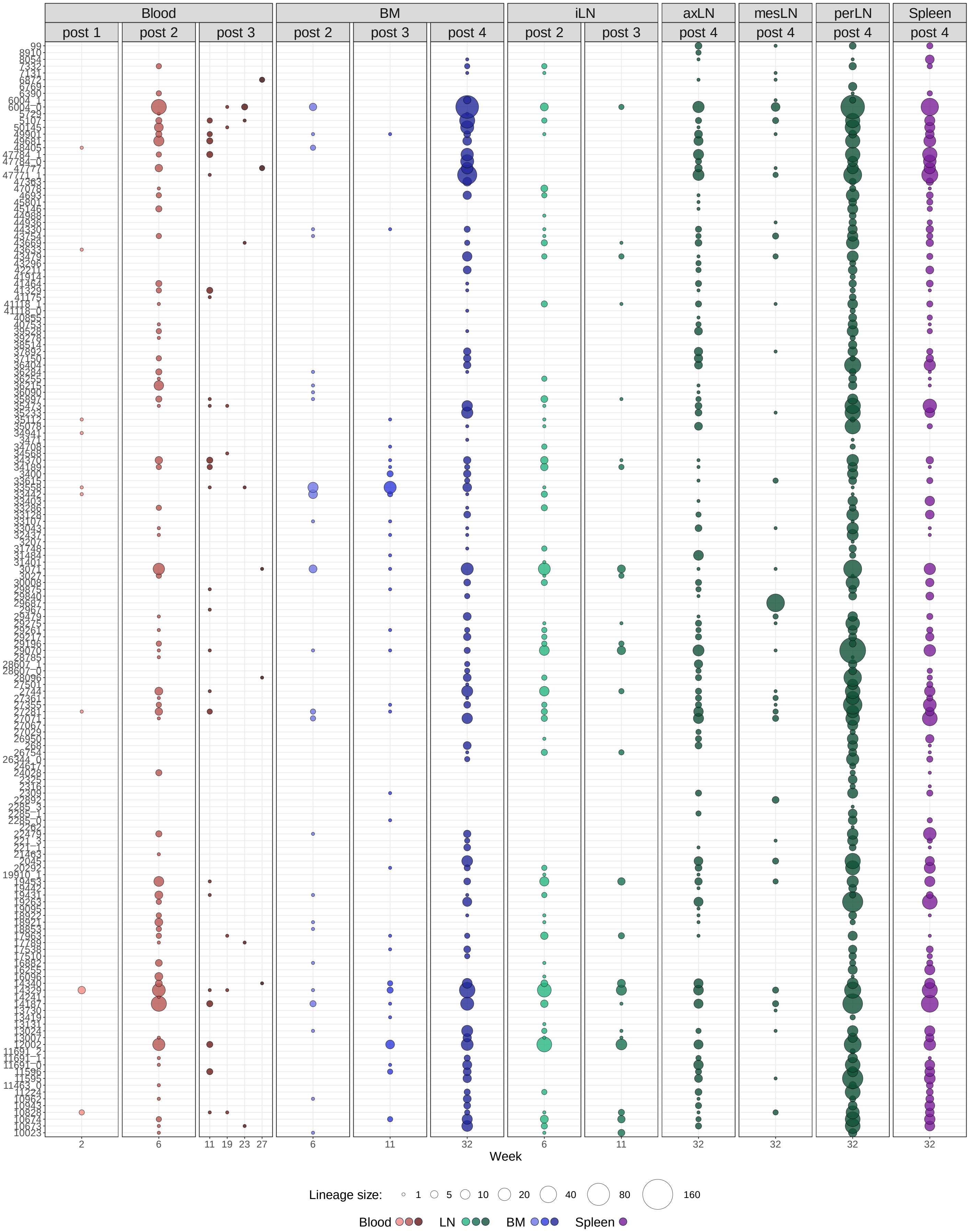

Supplementary Fig 3. Additional H03 queried lineages traced in different immune compartments. Dissemination of the additional queried lineages (not shown in Figure 3B) where the bubble size is directly proportional to the number of sequences identified for each lineage in each library. The x-axis of each section shows the sample compartment, grouped by time point with the sampling week indicated. Source data are provided as a Source Data file.

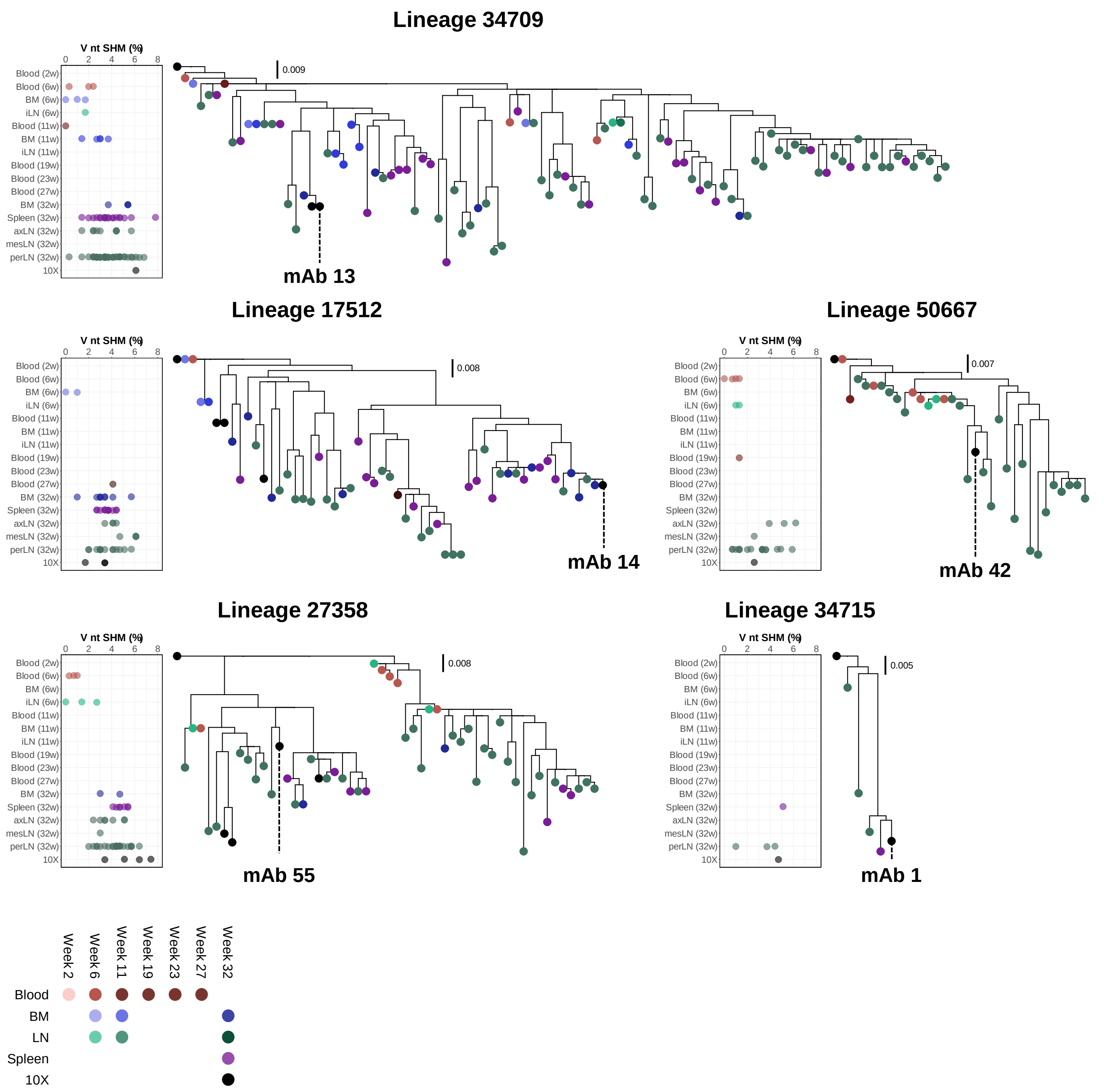

Supplementary Fig 4. Phylogenetic trees of additional neutralizing antibodies. Phylogenetic trees construction by maximum-likelihood of additional neutralizing mAbs. Traced members are displayed as dots colored in red, blue, green, and purple for blood, BM, LN, and spleen, respectively, with darker shades for increasing weeks. Members obtained from 10x single cell paired V(D)J sequencing are displayed as black dots. On the right side of every phylogenetic tree, a summary of V SHM of each tree's members is displayed as a dot plot following the same color scheme used in the tree. V SHM % at the nucleotide level is displayed on the x axis. Compartments of origin of each member are displayed on the y axis in temporal order.

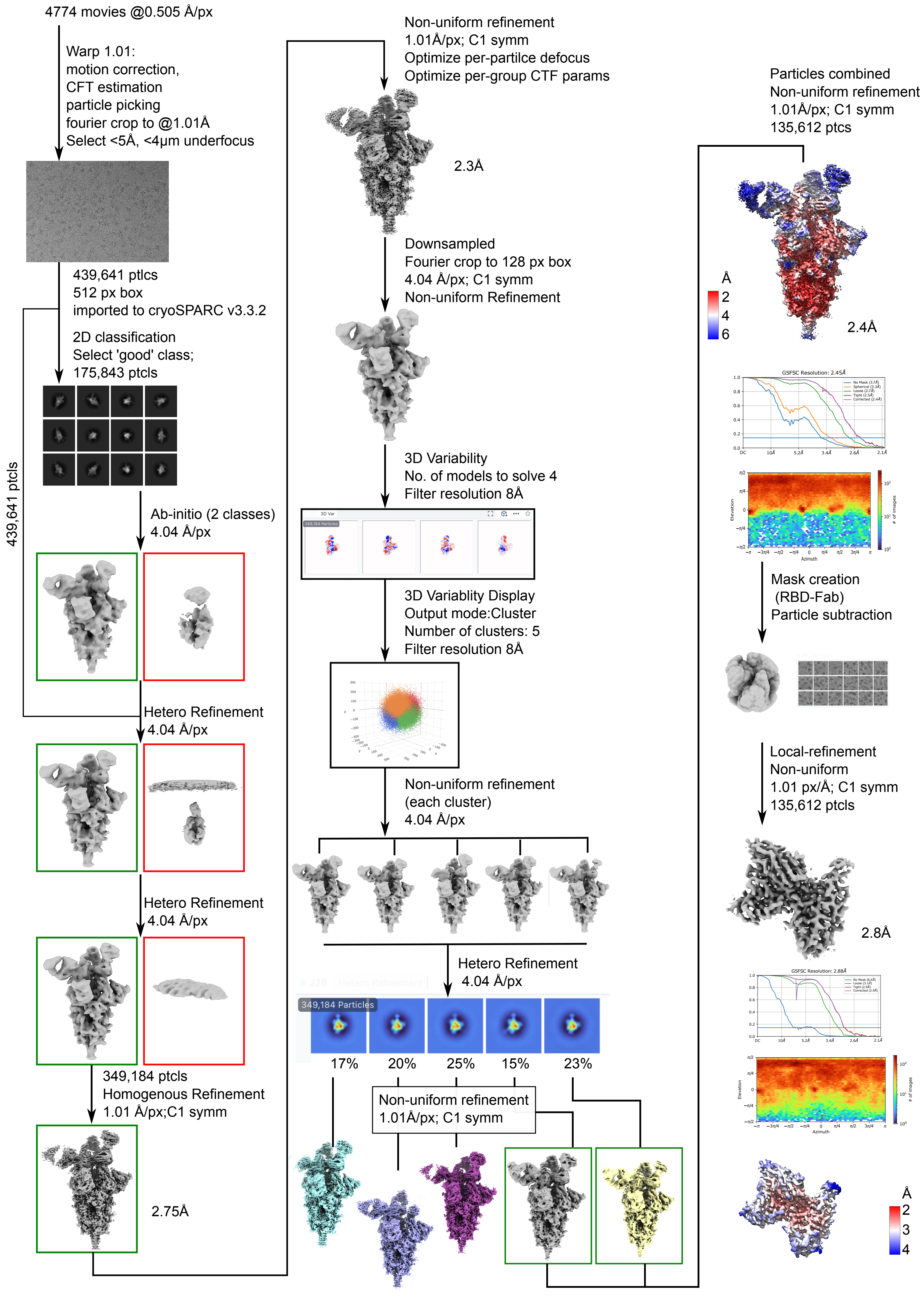

4774 movies @0.505 Å/px
Warp 1.01:
motion correction,
CFT estimation
particle picking
fourier crop to @1.01Å
Select <5Å, <4μm underfocus
439,641 ptlcs
512 px box
imported to cryoSPARC v3.3.2
439,641 ptcls
2D classification
Select 'good' class;
175,843 ptcls
Ab-initio (2 classes)
4.04 Å/px
Hetero Refinement
4.04 Å/px
Hetero Refinement
4.04 Å/px
349,184 ptcls
Homogenous Refinement
1.01 Å/px;C1 symm
2.75Å
Non-uniform refinement
1.01Å/px; C1 symm
Optimize per-partilce defocus
Optimize per-group CTF params
2.3Å
Downsampled
Fourier crop to 128 px box
4.04 Å/px; C1 symm
Non-uniform Refinement
3D Variability
No. of models to solve 4
Filter resolution 8Å
3D Var
349,184 Particles
3D Variablity Display
Output mode:Cluster
Number of clusters: 5
Filter resolution 8Å
Non-uniform refinement
(each cluster)
4.04 Å/px
Hetero Refinement
4.04 Å/px
349,184 Particles
17%
20%
25%
15%
23%
Non-uniform refinement
1.01Å/px; C1 symm
Particles combined
Non-uniform refinement
1.01Å/px; C1 symm
135,612 ptcs
Å
2
4
6
2.4Å
GSFSC Resolution: 2.45Å
No Mask (3.7Å)
Spherical (3.3Å)
Loose (2.7Å)
Tight (2.5Å)
Corrected (2.4Å)
Elevation
Azimuth
# of images
Mask creation
(RBD-Fab)
Particle subtraction
Local-refinement
Non-uniform
1.01 px/Å; C1 symm
135,612 ptcls
2.8Å
GSFSC Resolution: 2.88Å
No Mask (6.6Å)
Loose (3.1Å)
Tight (2.9Å)
Corrected (2.9Å)
Elevation
Azimuth
# of images
Å
2
3
4

Supplementary Fig 5. Cryo-EM processing. Particles were picked using Warp v1.0.1 and classified using CryoSPARC v3.3.2 for downstream refinement. Local reconstruction resolution was mapped to a locally sharpened map. FSC curve and Guinier plot are calculated by CryoSPARC v3.3.2.

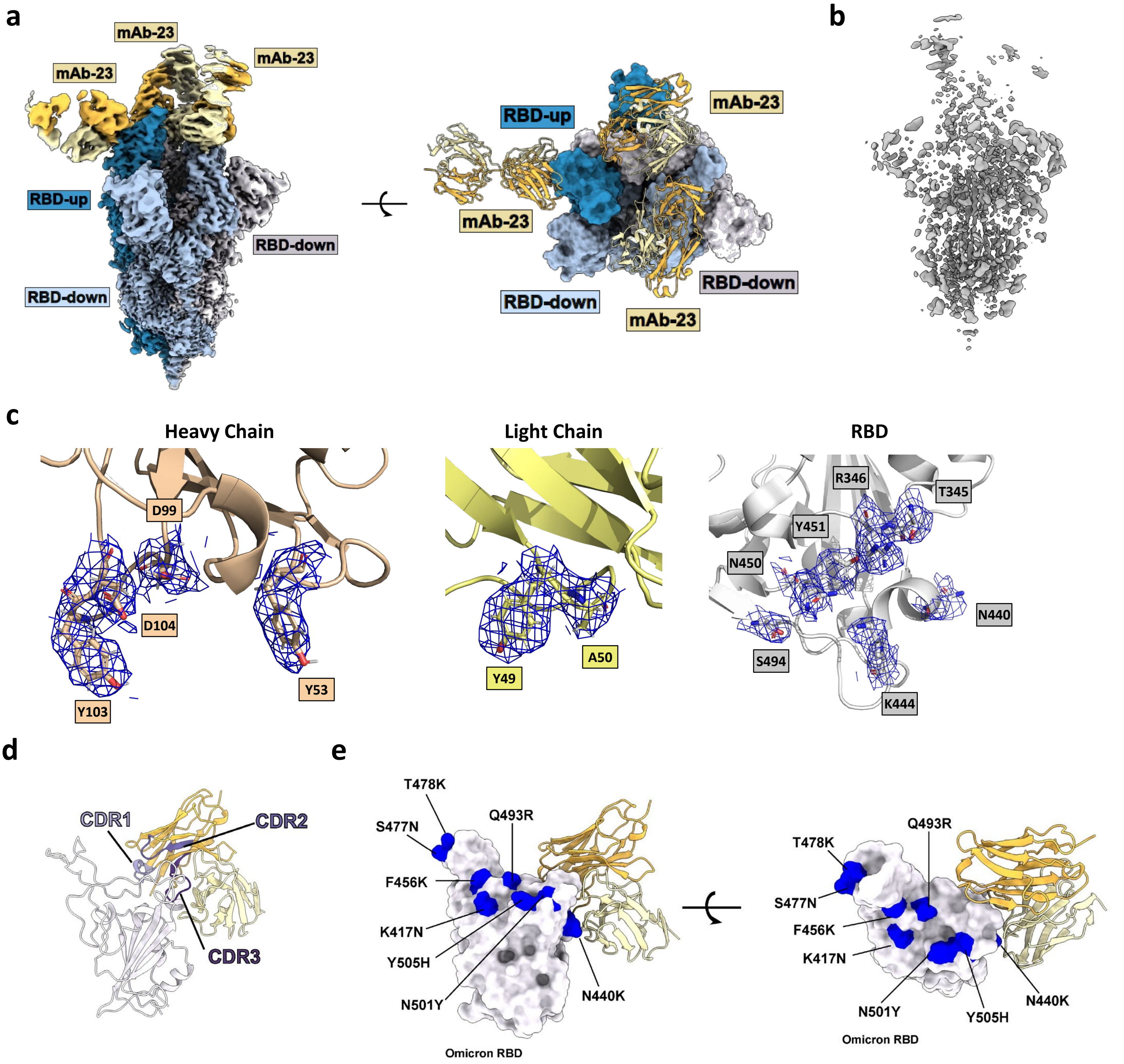

Supplementary Fig 6. Structure of mAb 23 in complex with the SARS-CoV-2 spike. a Lateral (left) and top (right) reconstruction of mAb 23 interaction with D614G-derived spike via cryo-EM. b. Full spike-mAb 23 complex omit map, prepared with CCP-EM Servalcat, contour level: 0.000103. **c** 2Fo-Fc Map electron density map of contact residues, contoured at 1 σ (blue mesh). Heavy chain paratope in orange (left), light chain paratope in yellow (center), and RBD epitope in grey (right). **d** Highlight of mAb 23 HCDR loops. **e** Mapping of common Omicron amino acids variant positions on the SARS-CoV-2 RBD.

Supplementary Table 1. NHP chain specific primers for 10X based single cell cDNA synthesis and amplification.

Primer	Sequence	Master Mix
10X_RM_IGHA_1	ACGTGGCATGTCACGGACTC	1
10X_RM_IGHD_1	CTGGCTGCTTGTCGTGTAGCTG	1
10X_RM_IGHE_1	TGTTGACCTCTTTGTCTGCGG	1
10X_RM_IGHG_1	TTGTCCACCTTGGTGTTGCT	1
10X_RM_IGHM_1	TACTTGCCCCCTCTCAGGACT	1
10X_RM_IGK_1	GTCCTGCTCTGTGACACTCTC	1
10X_RM_IGL_1	GTCTCCACTCCCGCGTTGAC	1
10X_RM_IGHA_2	TTGCTCCAGGTCACGTTGAGTG	2
10X_RM_IGHD_2	CAGGTGACAGTCACGGACTTTG	2
10X_RM_IGHE_2	GGCTGGTAAGGTCATAGTGCTT	2
10X_RM_IGHG_2	AGCCCTGAGGACTGTAGGA	2
10X_RM_IGHM_2	GCATTCTCACAGGAGACGAGG	2
10X_RM_IGK_2	ATTCAGCAGGCACACAACAGAG	2
10X_RM_IGL_2	AGACACACTAGTGTGGCCTTG	2

Supplementary Table 2. Library reads summary pre and post cleanup with FAD and Chimera detection.

Library	Total merged reads	Post-FAD	Post-CHIMERA	Chimeric %
H03-post1-blood	4692584	6327	6193	2.12
H03-post2-BM	3221169	16594	16140	2.74
H03-post2-iLN	3544903	16503	16098	2.45
H03-post2-blood	4590184	7890	7744	1.85
H03-post3-BM	5073720	19813	19240	2.89
H03-post3-iLN	3067779	3668	3508	4.36
H03-post3-blood	6479579	5329	5212	2.20
H03-post3.19w-blood	1727719	5000	4922	1.56
H03-post3.23w-blood	2567793	7734	7604	1.68
H03-post3.27w-blood	3738908	7009	6899	1.57
H03-post4-BM	4186761	13153	11465	12.83
H03-post4-axLN	1954733	7930	7703	2.86
H03-post4-mesLN	1077148	16044	15683	2.25
H03-post4-perLN	1472563	8607	8428	2.08
H03-post4-spleen	1435711	4560	4054	11.10
I10-post1-blood	4533604	9903	9655	2.50
I10-post2-BM	3008350	63569	61523	3.22
I10-post2-iLN	4457238	11514	11038	4.13
I10-post2-blood	4847225	4527	4390	3.03
I10-post3-BM	4397414	39471	38091	3.50
I10-post3-iLN	5273030	9804	9463	3.48
I10-post3-blood	4639468	6200	6049	2.44
I10-post3.19w-blood	2322594	4506	4406	2.22
I10-post3.23w-blood	2005041	4964	4851	2.28
I10-post3.27w-blood	2370272	4550	4408	3.12
I10-post4-BM	2652688	10120	9824	2.92
I10-post4-iLN-L	2309079	4416	4322	2.13
I10-post4-iLN-R	4159311	9604	9400	2.12
I10-post4-spleen	2580445	6243	6020	3.57
I10-post4-blood	1849560	5826	5666	2.75

Supplementary Table 3: Cryo-EM data collection, refinement, and validation statistics

	**SARS-CoV-2 Spike + Fab mAb 23 (EMDB: 18180) (PDB: 8Q5Y)**	**RBD + Fab mAb 23 (EMDB: 17451) (PDB: 8P5M)**
**Data collection and processing**		
**Magnification (nominal)**	165,000	
**Voltage (kV)**	300	
**Electron exposure (e–/Å^2)**	54.6	
**Underfocus range (µm)**	0.3–2	
**Pixel size (Å)**	0.505	
**Symmetry imposed**	C1	
**Initial particle images (no.)**	439,641	
**Final particle images (no.)**	135,612	135,612
**Map resolution (Å)**<br>FSC threshold	2.45<br>0.143	2.88<br>0.143
**Refinement**		
**Initial model used (PDB code)**	7A25	7A25
**Map sharpening *B* factor (Å^2)**	107.1	142.0
**Model composition**<br>Non-hydrogen atoms<br>Protein residues<br>Ligands	 30930<br>3873<br>52 NAG	 3260<br>419<br>1 NAG
***B* factors (Å^2)**<br>Protein<br>Ligand	 80.67<br>104.08	 84.9<br>-
**R.m.s. deviations**<br>Bond lengths (Å)<br>Bond angles (°)	 0.007<br>0.678	 0.005<br>1.042
**Validation**<br>MolProbity score[1]<br>Clashscore[1]<br>Poor rotamers (%)	 1.47<br>8.79<br>0.0	 1.37<br>3.60<br>0.0
**Ramachandran plot[2]**<br>Favored (%)<br>Allowed (%)<br>Disallowed (%)	 98.42<br>1.52<br>0.05	 96.61<br>3.39<br>0.0

Supplementary References

1. Chen, V. B. *et al.* MolProbity: All-atom structure validation for macromolecular crystallography. *Acta Crystallogr D Biol Crystallogr* (2010) doi:10.1107/S0907444909042073.
2. Ramachandran, G. N., Ramakrishnan, C. & Sasisekharan, V. Stereochemistry of polypeptide chain configurations. *J Mol Biol* **7**, 95–99 (1963).
