# Supplementary material for: Multi-compartmental diversification of neutralizing antibody lineages dissected in SARS-CoV-2 spike-immunized macaques
Source: Nat Commun. 2024 Jul 27;15:6338. doi: 10.1038/s41467-024-50286-0 (PMC11283548; doi:10.1038/s41467-024-50286-0)
Supplement: Supplementary file 3 — Description of Additional Supplementary Files [file 41467_2024_50286_MOESM3_ESM.pdf]

Description of Additional Supplementary Files

File name: Supplementary Data 1
Description: HC and LC V and J germline alleles identified with IgDiscover in H03 and I10. Both nucleotide sequences and translated amino acid sequences are reported.

File name: Supplementary Data 2
Description: Summary of single cell tracing status at paired VDJ level and lineage level.

File name: Supplementary Data 3
Description: Genetic information of spike-specific cloned mAbs.

File name: Supplementary Data 4
Description: Lineage 10644 phylogenetic tree SHM count for total VDJ sequence, V sequence and J sequence.

File name: Supplementary Data 5
Description: SARS-CoV-2 RBD amino acid variant frequencies based on Outbreak.info reports.
