# Supplementary material for: Multi-compartmental diversification of neutralizing antibody lineages dissected in SARS-CoV-2 spike-immunized macaques
Source: Nat Commun. 2024 Jul 27;15:6338. doi: 10.1038/s41467-024-50286-0 (PMC11283548; doi:10.1038/s41467-024-50286-0)
Supplement: Supplementary file 9 — Reporting Summary [file 41467_2024_50286_MOESM9_ESM.pdf]

nature portfolio

Corresponding author(s): Marco Mandolesi

Last updated by author(s): Jun 20, 2024

Reporting Summary

Nature Portfolio wishes to improve the reproducibility of the work that we publish. This form provides structure for consistency and transparency in reporting. For further information on Nature Portfolio policies, see our Editorial Policies and the Editorial Policy Checklist.

Statistics

For all statistical analyses, confirm that the following items are present in the figure legend, table legend, main text, or Methods section.

n/a	Confirmed	
☐	☒	The exact sample size (*n*) for each experimental group/condition, given as a discrete number and unit of measurement
☐	☒	A statement on whether measurements were taken from distinct samples or whether the same sample was measured repeatedly
☒	☐	The statistical test(s) used AND whether they are one- or two-sided<br>*Only common tests should be described solely by name; describe more complex techniques in the Methods section.*
☒	☐	A description of all covariates tested
☒	☐	A description of any assumptions or corrections, such as tests of normality and adjustment for multiple comparisons
☐	☒	A full description of the statistical parameters including central tendency (e.g. means) or other basic estimates (e.g. regression coefficient) AND variation (e.g. standard deviation) or associated estimates of uncertainty (e.g. confidence intervals)
☒	☐	For null hypothesis testing, the test statistic (e.g. *F*, *t*, *r*) with confidence intervals, effect sizes, degrees of freedom and *P* value noted<br>*Give P values as exact values whenever suitable.*
☒	☐	For Bayesian analysis, information on the choice of priors and Markov chain Monte Carlo settings
☒	☐	For hierarchical and complex designs, identification of the appropriate level for tests and full reporting of outcomes
☒	☐	Estimates of effect sizes (e.g. Cohen's *d*, Pearson's *r*), indicating how they were calculated

Our web collection on statistics for biologists contains articles on many of the points above.

Software and code

Policy information about availability of computer code

Data collection	CellRanger v3.1.0 was used to analyze the single cell sequencing output from 10X Chromium.<br>Data from cryo-EM were processed with the following using the following softwares: particles were picked using Warp v1.0.1 and classified using CryoSPARC v3.3.2 for downstream refinement.
Data analysis	IgDiscover22 v1.0.0 is available at https://gitlab.com/gkhlab/igdiscover22, Scripts used to generate all results in the paper are available at: https://gitlab.com/gkhlab/Multi-compartmental_diversification_of_neutralizing_antibody_lineages_dissected_in_SARS-CoV-2_spike-immunized_macaques. The HMM code for chimera identification is available at https://github.com/MurrellGroup/CHMMera/.<br>Cryo-EM structure refinement and manual model building were performed using COOT v0.9.8.9173 and PHENIX v1.20.

For manuscripts utilizing custom algorithms or software that are central to the research but not yet described in published literature, software must be made available to editors and reviewers. We strongly encourage code deposition in a community repository (e.g. GitHub). See the Nature Portfolio guidelines for submitting code & software for further information.

Data

Policy information about availability of data

All manuscripts must include a data availability statement. This statement should provide the following information, where applicable:

- Accession codes, unique identifiers, or web links for publicly available datasets
- A description of any restrictions on data availability
- For clinical datasets or third party data, please ensure that the statement adheres to our policy

Repertoire sequencing data have been deposited in the European nucleotide archive (ENA) with the following accession numbers: from ERR12544449 to ERR12544478.
Monoclonal antibody sequences are deposited at Genbank with the following accession numbers: from PP208826 to PP208901.
The associated accession numbers, coordinates and structure factors of the cryo-EM data reported in this paper are available from the Protein Data Bank (PDB) with accession code PDB: 8Q5Y.

Research involving human participants, their data, or biological material

Policy information about studies with human participants or human data. See also policy information about sex, gender (identity/presentation), and sexual orientation and race, ethnicity and racism.

Reporting on sex and gender	*Use the terms sex (biological attribute) and gender (shaped by social and cultural circumstances) carefully in order to avoid confusing both terms. Indicate if findings apply to only one sex or gender; describe whether sex and gender were considered in study design; whether sex and/or gender was determined based on self-reporting or assigned and methods used. Provide in the source data disaggregated sex and gender data, where this information has been collected, and if consent has been obtained for sharing of individual-level data; provide overall numbers in this Reporting Summary. Please state if this information has not been collected. Report sex- and gender-based analyses where performed, justify reasons for lack of sex- and gender-based analysis.*
Reporting on race, ethnicity, or other socially relevant groupings	*Please specify the socially constructed or socially relevant categorization variable(s) used in your manuscript and explain why they were used. Please note that such variables should not be used as proxies for other socially constructed/relevant variables (for example, race or ethnicity should not be used as a proxy for socioeconomic status). Provide clear definitions of the relevant terms used, how they were provided (by the participants/respondents, the researchers, or third parties), and the method(s) used to classify people into the different categories (e.g. self-report, census or administrative data, social media data, etc.) Please provide details about how you controlled for confounding variables in your analyses.*
Population characteristics	*Describe the covariate-relevant population characteristics of the human research participants (e.g. age, genotypic information, past and current diagnosis and treatment categories). If you filled out the behavioural & social sciences study design questions and have nothing to add here, write "See above."*
Recruitment	*Describe how participants were recruited. Outline any potential self-selection bias or other biases that may be present and how these are likely to impact results.*
Ethics oversight	*Identify the organization(s) that approved the study protocol.*

Note that full information on the approval of the study protocol must also be provided in the manuscript.

Field-specific reporting

Please select the one below that is the best fit for your research. If you are not sure, read the appropriate sections before making your selection.

☒ Life sciences ☐ Behavioural & social sciences ☐ Ecological, evolutionary & environmental sciences

For a reference copy of the document with all sections, see nature.com/documents/nr-reporting-summary-flat.pdf

Life sciences study design

All studies must disclose on these points even when the disclosure is negative.

Sample size	Samples from two macaques were utilized for lineage tracing. The number of macaques was based off of plasma readout published in Mandolesi et al, 2021 (ref) and of animal availability.
Data exclusions	V(D)J reads from single cell sequencing were filtered using the following procedure. The filtered V(D)J contigs files were then assigned to individualized H03 or I10 IGH, IGK, and IGL database. Non-productive sequences or sequences without CDR3 identification were removed. Subsequently, we filtered cells with multiple IGH, IGK or IGL assignments and cells with presence of both IGK and IGL. Finally, we set a strict threshold for hashing barcode assignment. The sequences with maximum value of counts per hash lower than 20 were classified as “Low_counts”. Hashes accounting for >= 60% of all counts were selected for assignment. Otherwise, the sequences were classified as “Unassigned”. Furhtermore, V(D)J sequences from single cell analysis were initially assigned to a compartment via TotalSeq-C hashing barcodes count. With counts equal to or over 20, a threshold of 60% was used to determine reliable assignment to one compartment. The

	rest of the sequences were divided into two groups, low_count and unassigned, based on TotalSeq-C count lower than 20 or failure to pass the 60% threshold, respectively. To determine whether these sequences belonged to H03 or I10, they were assigned to each animal individualized databases and underwent lineage tracing analysis. Only sequences traced to lineages within only one animal were retained and used for downstream analysis in this study.
Replication	Antibody binding and neutralization assays readouts were produced in triplicates in each experiment.
Randomization	No randomization was used in this study design.
Blinding	No blinding was used in this study design.

Reporting for specific materials, systems and methods

We require information from authors about some types of materials, experimental systems and methods used in many studies. Here, indicate whether each material, system or method listed is relevant to your study. If you are not sure if a list item applies to your research, read the appropriate section before selecting a response.

Materials & experimental systems

n/a	Involved in the study
☐	☒ Antibodies
☐	☒ Eukaryotic cell lines
☒	☐ Palaeontology and archaeology
☐	☒ Animals and other organisms
☒	☐ Clinical data
☒	☐ Dual use research of concern
☒	☐ Plants

Methods

n/a	Involved in the study
☒	☐ ChIP-seq
☐	☒ Flow cytometry
☒	☐ MRI-based neuroimaging

Antibodies

Antibodies used	ELISA:<br>- Goat anti-Human IgG(H+L)-HRP (Cat#2015-05) from Southern Biotech<br>FACS:<br>- Mouse anti-Human CD3ε FITC (Cat# 556611) Clone SP34 from BD Pharmingen™<br>- Mouse anti-Human CD14 BV786 (Cat# 563698) Clone M5E2 from BD Horizon™<br>- Mouse anti-Human CD20 BV421 (Cat# 562873) Clone 2H7 from BD Horizon™
Validation	All antibodies were commercial, obtained from BD Biosciences and Southern Biotech companies, which verify the reactivity of their antibodies. These antibodies were not validated internally, as we relied on quality control checks performed by the manufacturer.<br>- Goat anti-Human IgG(H+L)-HRP (Cat#2015-05) from Southern Biotech. Lot# C0517-N921D. According to manufacturer, Quality tested applications for relevant formats include ELISA and FLISA. ELISA plate was coated with purified human IgG, IgM, and IgA. Immunoglobulins were detected with serially diluted antibody.<br>- Mouse anti-Human CD3ε FITC (Cat# 556611) Clone SP34 from BD Pharmingen™. Lot# 0073812. According to manufacturer, routinely tested for Flow cytometry application.<br>- Mouse anti-Human CD14 BV786 (Cat# 563698) Clone M5E2 from BD Horizon™. Lot# 0209397. According to manufacturer, routinely tested for Flow cytometry application.<br>- Mouse anti-Human CD20 BV421 (Cat# 562873) Clone 2H7 from BD Horizon™. Lot# 0314110. According to manufacturer, routinely tested for Flow cytometry application.

Eukaryotic cell lines

Policy information about cell lines and Sex and Gender in Research

Cell line source(s)	FreeStyle™ 293-F Cells (ThermoFisher Scientific Cat# R79007) (Human female origin) cell lines were utilized in this study to produce mAbs, spike, and RBD recombinant proteins.
Authentication	FreeStyle™ 293-F Cells (ThermoFisher Scientific Cat# R79007) were not authenticated internally.
Mycoplasma contamination	Cell lines tested negative for mycoplasma contamination.
Commonly misidentified lines<br>(See ICLAC register)	No commonly misidentified cell lines were used.

Animals and other research organisms

Policy information about studies involving animals; ARRIVE guidelines recommended for reporting animal research, and Sex and Gender in Research

Laboratory animals	One male (I10) and one female (H03) rhesus macaque (Macaca mulatta) of Chinese origin, 4-5 years old, were housed at the Astrid Fagraeus Laboratory at Karolinska Institutet. Housing and care procedures complied with the provisions and general guidelines of the Swedish Board of Agriculture. The facility has been assigned an Animal Welfare Assurance number by the Office of Laboratory Animal Welfare (OLAW) at the National Institutes of Health (NIH). The macaques were housed in groups in 14 m3 enriched cages. They were habituated to the housing conditions for more than six weeks before the start of the experiment and subjected to positive reinforcement training to reduce the stress associated with experimental procedures. The macaques were weighed at each sampling. All animals were confirmed negative for simian immunodeficiency virus (SIV), simian T cell lymphotropic virus, simian retrovirus type D and simian herpes B virus.
Wild animals	This study did not involve wild animals.
Reporting on sex	Single cell sequencing, NGS sequencing, and lineage tracing analysis apply for both one male (I10) and one female (H03) rhesus macaque (Macaca mulatta) of chinese origin. MAbs isolation and phylogenetic analysis apply to B cell lineage from one female (H03) rhesus macaque. Sex-based analysis was not performed due to the low number of representative animals per sex and due to the study design.
Field-collected samples	This study did not involve samples collected from the field.
Ethics oversight	The animal work was conducted with the approval of the regional Ethical Committee on Animal Experiments (Stockholms Norra Djurförsöksetiska Nämnd). All animal procedures were performed according to approved guidelines, following permits #18427-2019 and #10895-2020.

Note that full information on the approval of the study protocol must also be provided in the manuscript.

Plants

Seed stocks	*Report on the source of all seed stocks or other plant material used. If applicable, state the seed stock centre and catalogue number. If plant specimens were collected from the field, describe the collection location, date and sampling procedures.*
Novel plant genotypes	*Describe the methods by which all novel plant genotypes were produced. This includes those generated by transgenic approaches, gene editing, chemical/radiation-based mutagenesis and hybridization. For transgenic lines, describe the transformation method, the number of independent lines analyzed and the generation upon which experiments were performed. For gene-edited lines, describe the editor used, the endogenous sequence targeted for editing, the targeting guide RNA sequence (if applicable) and how the editor was applied.*
Authentication	*Describe any authentication procedures for each seed stock used or novel genotype generated. Describe any experiments used to assess the effect of a mutation and, where applicable, how potential secondary effects (e.g. second site T-DNA insertions, mosiacism, off-target gene editing) were examined.*

Flow Cytometry

Plots

Confirm that:

- [x] The axis labels state the marker and fluorochrome used (e.g. CD4-FITC).
- [x] The axis scales are clearly visible. Include numbers along axes only for bottom left plot of group (a 'group' is an analysis of identical markers).
- [x] All plots are contour plots with outliers or pseudocolor plots.
- [x] A numerical value for number of cells or percentage (with statistics) is provided.

Methodology

Sample preparation	PBMCs were isolated by density-gradient centrifugation from EDTA blood by Ficoll-Paque PLUS (GE Healthcare), washed extensively in PBS, and treated with red blood cell lysis buffer. Single-cell suspensions of splenocytes and LN cells were prepared using a 70-µm cell strainer and a syringe plunger. Cell suspensions were washed once in Ca2+- and Mg2+-free PBS (Sigma-Aldrich) and treated with red blood cell lysis buffer. Cell samples collected from different immune compartments were frozen in 90% heat-inactivated FBS and 10% DMSO (Sigma-Aldrich) and stored at -80°C. Prior to staining, cryo-preserved cells were thawed in a water bath at 37°C. Dead cells were discriminated using a LIVE/DEAD™ Fixable Aqua Dead Cell Stain Kit (Cat# L34957), following the manufacturer's protocol. To block nonspecific antibody binding to Fc receptors, cells were incubated with Fc Receptor Binding Inhibitor (eBioscience cat#14-9165-42) in PBS-2% FBS (FACS buffer) at 4°C for 30 min. Markers were then stained with fluorochrome-conjugated monoclonal antibodies at 4°C for 30 min. Finally, the spike probe was added to the stained cell suspension at a final concentration of 4 µg/ml. The stained cells were washed with 10 ml of prechilled PBS and resuspended in 1ml-500 µl of FACS buffer, passed through a 70-µm cell mesh (BD Biosciences).

Instrument	BD FACSAria™ Fusion
Software	- BD FACSDiva™ (v9.0.1) to collect the data and sort spike+ B cells prior to single cell sequencing.<br>- FlowJo (v10.8.1) to analyze and visualize the FACS gating strategy.
Cell population abundance	Purity was determined by specific and relevant staining with markers by flow cytometry. Spike+ B cell abundance from total B cells were the following: 0.44% for H03 iLN-R1, 0.91% for H03 iLN-R2, 0.48% for H03 LNOther, 0.63% for H03 Spleen and, 1.33% for I10 blood.
Gating strategy	Lymphocytes population were identified via SSC-A/FSC-A discrimination on singlet cells identified via FSC-H/FSC-A. Then, live cells were identified as BV510 negative by staining with LIVE/DEAD™ Fixable Aqua Dead Cell Stain Kit (Cat# L34957). Afterwards, B cells were identified as CD3-/CD14-/CD20+ using CD3-FITC, CD14-BV786 and CD20-BV421. Finally, Spike+ B cells were identified with a spike-APC probe.

☒ Tick this box to confirm that a figure exemplifying the gating strategy is provided in the Supplementary Information.
